# Changes in Self-Rated Health Right After Immigration: A Panel Study of Economic, Social, Cultural, and Emotional Explanations of Self-Rated Health Among Immigrants in the Netherlands

**DOI:** 10.3389/fsoc.2019.00045

**Published:** 2019-06-06

**Authors:** Marcel Lubbers, Mérove Gijsberts

**Affiliations:** ^1^Netherlands Interdisciplinary Demographic Institute (NIDI/KNAW), The Hague, Netherlands; ^2^Department of Sociology, Radboud University Nijmegen, Nijmegen, Netherlands; ^3^University of Groningen, Groningen, Netherlands; ^4^Netherlands Institute for Social Research, The Hague, Netherlands; ^5^ASW: Cultural Diversity and Youth, Utrecht University, Utrecht, Netherlands

**Keywords:** recent immigrants, immigrant health, healthy immigrant effect, discrimination, homesickness

## Abstract

Immigrants are often found to rate their health better than the native population does. It is, however, suggested that this healthy immigrant effect declines with an enduring length of stay. With Dutch panel data, we investigate which patterns in self-rated health can be found among immigrants shortly after their migration. We test to what extent economic, social, cultural and emotional explanations affect the changes that immigrants report in self-rated health. Based on a four-wave panel, our results support the immigrants' health decline hypothesis, since the self-rated health decreases in the first years after immigration to the Netherlands. The major change occurs between immigrants rating their health no longer as “very good,” but as “good.” Shortly after immigration, self-rated health is associated with being employed and a higher income. Hazardous work and physically heavy work decrease self-rated health. Notwithstanding these effects, social, cultural, and emotional explanations turn out to be stronger. A lack of Dutch friends, perceptions of discrimination, perceived cultural distance, and feelings of homesickness strongly affect self-rated health. Furthermore, in understanding changes in self-rated health, the effects of making contact with Dutch people and changes in the perception of discrimination are definitive. However, contact with Dutch people did not decrease and discrimination did not increase over time, making them ineligible as an explanation for overall health decrease. Only the small effect that first-borns have may count as a reason for decreased self-rated health, since many of the recent immigrants we followed started families in the first years after immigration. Our findings leave room for the coined “acculturation to an unhealthier lifestyle thesis,” and we see promise in a stronger focus on the role of unmet expectations in the first years after immigration.

## Introduction

It is a paradox that has been found over and again; notwithstanding immigrants' lower socio-economic position on arrival, they turn out to be healthier than the receiving population (John et al., 2012; Urquia et al., 2012). This Healthy Immigrant Effect is due to selection effects, i.e., healthier immigrants are more likely to migrate (Jasso et al., 2004; Wallace and Kulu, 2014; Riosmena et al., 2017). Predominantly, people who have energy and ambition are likely to migrate and are, therefore, in better health compared to the general population (see Antecol and Bedard, 2006; Singh Setia et al., 2011; Kennedy et al., 2015).

If immigrants are indeed a selection of healthy people (Farré, 2016; Riosmena et al., 2017), the question arises as to why it is often found that immigrant populations in general (including those living for an enduring period in the resident country), and especially those from non-Western origins, often rate their health as worse than the native population (see the systemic review from Nielsen and Krasnik, 2010; e.g., Solé-Auró and Crimmins, 2008; Wengler, 2011; Moullan and Jusot, 2014; Jatrana et al., 2018). It might be that very different groups who arrived in different periods with various socio-economic positions are compared (as given as a possible explanation by Jasso et al., 2004). Yet it might also be that health conditions deteriorate because of the migration experience, since many studies support a negative association between “length of stay” and health perceptions (Jasso et al., 2004). Mainly US and Canadian longitudinal research support this immigrant health decline hypothesis, with convergence to lower levels of health with enduring length of stay or over generations (McDonald and Kennedy, 2004; Newbold, 2005; Antecol and Bedard, 2006; Acevedo-Garcia et al., 2010; Goldman et al., 2014), although recent panel-studies from Lu et al. (2017) and Jatrana et al. (2018) refute a decline in reported health among (established) immigrants to the US and Australia, respectively, and report stability. A systematic review of studies in Canada states that, “The healthy immigrant effect is stronger for recent …. immigrants and vanishes among more established immigrants. However, it is not possible to determine if these *duration effects* reflect true convergence or overshoot because the majority of the studies were based on cross-sectional analyses.” (Vang et al., 2015, p.1; Vang et al., 2017). In Europe as well, there is discussion on the association between length of stay and self-rated health, with many studies supporting a negative association (Huijts and Kraaykamp, 2012; Rechel et al., 2013; Vacková and Brabcová, 2015), whereas others even find that immigrants who have a shorter length of stay report lower levels of health (Leão et al., 2009). Additionally, here it is claimed that the field would benefit from studies following immigrants over time (De Valk and Fokkema, 2018; Jatrana et al., 2018).

In this article we study self-rated health of immigrants in the first years after immigration, in line with the recommendations from Jasso et al. (2004) to trace immigrants' acculturation trajectories from right after immigration. The importance of studying the health status of immigrants cannot be stressed enough. Not only is health crucial for obtaining an economically stable position for the growing immigrant populations, but it also is essential for the wellbeing of the children of immigrants, as well as for societies as a whole, since the costs of the health sector make up the major shares of government spending. By employing a four-wave panel, the better insight needed into the dynamics of health after immigration can be obtained (Rechel et al., 2013). We study changes in self-rated health among recent immigrants to the Netherlands from four different origin countries (excluding a country where mainly refugees migrated from), extending the literature on health dynamics after immigration to another country than the often-studied situation in the US, Canada or Australia (Kennedy et al., 2015). Most studies have applied a design to merely support or refute the immigrant health decline hypothesis; sometimes by conditionally testing on cohorts or relevant socio-structural characteristics. These studies show quite some variance in immigrant health decline depending on e.g., ethnic group and gender (Singh Setia et al., 2011; Urquia et al., 2012; Barbieri, 2016). These studies focus less on explanations of immigrant health decline (Jatrana et al., 2018). Following suggestions of Jasso et al. (2004) to integrate explanations of immigrant health decline from different domains, and Riosmena et al. (2017) and Kwak (2016, 2018) to get a more nuanced understanding of what contributes to changes in self-rated health, we seek explanations in immigrants' changes in the economic, social, and cultural domain. These domains have been suggested before to understand health differences (Venema et al., 1995; De Maio and Kemp, 2010; Nielsen and Krasnik, 2010), but were not applied simultaneously for understanding dynamics in self-rated health. When immigrants settle in a new country, the initial advantaged health position may diminish by the sometimes stressful economic (e.g., hardship in finding a job), social (e.g., lack of social contacts), and cultural (e.g., discrimination) experiences they have in their new country. Also, the emotional sphere may play a role (e.g., homesickness). If we do find support for a decline in self-rated health in the first years after immigration, we examine which of these explanations are most decisive in explaining the downward trend. We aim at four immigrant groups with rather different reasons for immigration and with different socio-economic positions in their receiving country: Bulgarian, Polish, Spanish and Turkish immigrants.

### Bulgarian, Polish, Spanish, and Turkish Immigrants in the Netherlands

As with most of the Western-European countries, the Netherlands recent history of immigration is characterized by labor migration, family migration, migration from former colonies, and asylum migration. Higher levels of immigration took off in the 1960s, when the booming economy resulted in labor shortage, and workers were recruited first from southern Europe (including Spain) and later from Turkey and Morocco. Whereas, the majority of southern European immigrants returned in the 1970s to their country of origin, the majority of Turkish and Moroccan immigrants stayed in Europe. The family reunification that followed made these immigrant groups the largest in the Netherlands. Taking the first and second generation together, Dutch citizens with a Turkish background comprise 2.3% of population; also Dutch citizens with a Moroccan background comprise 2.3% of the Dutch population (Statistics Netherlands, 2018). In 2017, almost a quarter of the Dutch population had an immigrant background; 11% of the population was registered as (first generation) immigrant and 12% as second generation (Statistics Netherlands, 2018). Immigrants from former Dutch colonies (Suriname, Dutch Antilles, Indonesia) take up a relevant share as well as refugees from Afghanistan, Iran, Iraq and Somalia, and more recently from Syria and Eritrea. The sharpest increase in immigrants in the last two decades came from Eastern Europeans. Due to enlargement of the European Union, Eastern Europeans obtained the right to freely move within the EU. In less than 10 years, the Polish immigrant community has become the sixth largest in the Netherlands. Also, immigration from countries like Bulgaria and Romania increased strongly. When in 2008 the international economic crisis hit Southern Europe hard, immigration from Spain and other Mediterranean countries also rose. The data in this study are from a panel study that started in 2013 and targeted newly arrived immigrants. Four groups with sizeable immigration figures in 2012 and 2013 were chosen: Polish, Bulgarian, Spanish and Turkish immigrants. No refugee group was included, since the number of refugees entering the country was small at the time. Studies on the selected immigrant populations show that Poles came almost solely for work reasons and almost all succeeded in finding jobs, albeit at lower levels than their educational credentials merit (Gijsberts and Lubbers, 2013). Bulgarian immigrants turned out to be more diverse; they came either for economic or study reasons. Moreover, among the Bulgarian immigrants there was a sizable Turkish Bulgarian minority and large variety in educational level (Engbersen et al., 2011). Spanish immigrants were mostly higher educated, searched for better job opportunities in the Netherlands and often found employment in ICT or universities (Gijsberts et al., 2016). Turkish immigrants were the only ones without the right of free movement to the Netherlands. By far, the majority of the Turkish immigrants came as family migrants, since they married a Dutch partner; a small share came to the Netherlands as student (Gijsberts and Lubbers, 2013).

## Expectations

Scholarly attention on immigrants' health decline suggests that immigrants may seem healthier at immigration but underreport health problems. One reason for this would be that immigrants are not diagnosed yet, since they under-utilize medical care in their new destination country. Both McDonald and Kennedy (2004) and Antecol and Bedard (2006) criticize this explanation, since it implies there is a serious increase in unknown health problems at the time of immigration. The origin countries in this study have advanced health care systems and it may be reasonable to expect that existing health problems would have been diagnosed earlier. Antecol and Bedard (2006) see more merit in acculturation explanations; the extent to which immigrants adopt the life-styles of the host-society. They convincingly show that an increase in BMI with enduring length of stay—as a result of adapting to the American lifestyle—is associated with worse subjective, as well as objective, health. Following the idea that the situation after immigration and changes in that situation are relevant for health assessment, we expect changes over time in self-rated health to be related to changes in the economic, social and cultural situation after immigration. All these aspects may influence homesickness, which we disentangle as emotional factor, which additionally may be a reason for changes in self-rated health. With the inclusion of these explanations simultaneously (Nielsen and Krasnik, 2010), we test a more comprehensive dynamic model and complement the theoretical models that mainly focus on assimilationist acculturation strategies to understand changes in immigrants' health (Abraído-Lanza et al., 2006; De Maio and Kemp, 2010; Acevedo-Garcia et al., 2012).

### Economic Domain

Most studies show a positive association between socio-economic status and (immigrants') health or health perceptions (Reijneveld, 1998; Brussaard et al., 2001; Wiking et al., 2004; McDonough et al., 2010; Wengler, 2011; Huijts and Kraaykamp, 2012; Alcántara et al., 2014). In some studies, the socio-economic gradient in immigrants' health perceptions is explained by the larger social network that comes with higher socio-economic status (Fokkema and Naderi, 2013). In other work, explanations are sought in the means it provides to sustain contacts in the country of origin and to receive approval by meeting expectations to send remittances (Dito et al., 2017). Mostly, a more direct effect from socio-economic status is expected. Employment and a sufficient income provide stability and reduce uncertainty, whereas unemployment does the opposite and is found to be associated with lower self-rated health (Huijts and Kraaykamp, 2012). Immigrants often face difficulties to enter the labor market and to find jobs fitting their educational skills (Amuedo-Dorantes and De la Rica, 2007; Kogan, 2011); facing these barriers over an extended period may reduce self-rated health. However, immigrants are thought to improve their socio-economic position with enduring stay (Chiswick et al., 2005; Akresh, 2008; Lubbers and Gijsberts, 2016). This socio-economic integration perspective on length of stay does not offer an explanation for the immigrant health decline; to the contrast, rising labor market participation and increasing income with longer residence in the country should lead to be better self-rated health, not lower health rating after immigration as is found so often (Antecol and Bedard, 2006). Economic integration, as measured by labor market participation, may however disguise the uncertainty or unfavorable work conditions that immigrants often face (Akresh, 2008). Immigrants who experience unemployment spells, who work in temporary contracts, who work irregular hours, and those doing physically hard work and hazardous work are likely to report less health with a longer stay (Gotsens et al., 2015). With the flexibilization of the labor market, immigrants may have encountered such insecurities more often with enduring stay, possibly affecting their health in a negative way (Rellstab et al., 2016).

### Social Domain

In the social domain, immigrant integration literature assumes that with a longer time of stay in the receiving country, immigrants expand their social network (Martinovic et al., 2008). Reduction of loneliness, by way of obtaining social contacts, will positively affect self-rated health (Hawkley et al., 2003; Cacioppo et al., 2010; Fokkema and Naderi, 2013; Tegegne, 2018). Social support and/or social capital is theorized and found to be highly relevant for well-being (Arpino and de Valk, 2018), lowering loneliness (De Jong Gierveld et al., 2015), and increasing self-rated health (Finch and Vega, 2003; Riosmena et al., 2017). But again, the trend to better social integration with longer length of stay (Sand and Gruber, 2018) is unlikely to explain a decline in health. Similarly, it is often assessed that in the first years after immigration family reunion takes places, with partners (and children) joining the immigrant, or with new union formations (Massey, 1990). It is unlikely that this will reduce the immigrants' health perception. Immigrants, being often relatively young and starting families, will be more likely to give rise to newborns. From research on the role of children on happiness and life satisfaction it is suggested that a first child reduces these subjective assessments (Stanca, 2012; Pollmann-Schult, 2014), although studies on this association are not conclusive (Myrskylä and Margolis, 2014), and it is not tested on recent immigrants. If newborns reduce happiness, and happiness is associated to self-rated health, it may explain decreasing self-rated health.

### Cultural Domain

Discrimination or acculturation stress is found to play a role in immigrants' health, with a lower health among immigrants who perceive discrimination of their origin group (Utsey et al., 2000; Finch and Vega, 2003; Mossakowski, 2003; Verkuyten, 2008; Safi, 2009; Abdulrahim et al., 2012; Huijts and Kraaykamp, 2012). Immigrants may be positively selected with favorable attitudes about their destination country before and just after immigration, they may develop a more realistic perception of the country of destination over time. Moreover, in the receiving country, they may perceive negativity toward immigrants in general and toward their country of origin in particular (McGinnity and Gijsberts, 2016). In Canada, De Maio and Kemp (2010) found that perceptions of discrimination are among the key explanations to understand deteriorating health assessment: immigrants who experienced discrimination were more likely to deteriorate in health; however, the study did not show whether a change in discrimination is associated with a change in health over time. Discrimination is thought to reduce people's feelings of confidence and acceptance, with consequences on happiness, self-rated health, and loneliness (Visser and El Fakiri, 2016). Immigrants may also become aware, or perceive stronger differences, between their country of origin culture and the receiving country's culture. A perceived larger cultural difference between origin and destination may also induce perceptions of non-belonging, uneasiness in the new environment, and loneliness (Klok et al., 2017), and may result in lower levels of happiness and consequently in lower self-rated health. Van Tilburg and Fokkema (2018) suggest that negative interpretations of social position are of key importance to understanding immigrants' well-being. We expect that over time, perceptions of discrimination and of cultural distance between the country of origin and destination increase, and that changes toward stronger perceptions of discrimination and more cultural distance are associated with lower self-rated health.

### Emotional Domain

Another explanation that connects to the social domain, but also can be motivated by the other domains we disentangle, is that of homesickness (Tartakovsky, 2007). Homesickness is found to be associated with lower levels of self-rated health (Van Tilburg et al., 1999) and is seen as “mini-grief,” that negatively affects well-being (Stroebe et al., 2002). Immigrants, generally, have left family behind, and although technological advancements have made contact with beloved ones easier than ever, we expect that missing friends and family increases with enduring stay, increasing levels of homesickness. Homesickness may increase or diminish with the success of establishing other social contacts in the country of residence (Tartakovsky, 2007) and may also be alleviated by favorable experiences in the economic domain. Feelings of non-belonging, instigated by the receiving population's attitudes, and coming to the fore in perceived group discrimination, is also found to instill homesickness (Watt and Badger, 2009). We expect that homesickness increases with longer stay and that an increase in homesickness is associated with lower self-rated rated health.

## Data and Measurements

We rely on a four-wave panel collected among newly registered immigrants to the Netherlands in 2012 and 2013, from Bulgaria, Poland, Spain and Turkey (Lubbers et al., 2018b). Immigrants over 18 who registered up to one and a half year before the start of the data collection were sampled (from the immigrants from Poland this was a random sample, from the other groups the whole population was approached). Immigrants were approached in their country of origin language and were sent a copy of the questionnaire as well as login-codes to offer the opportunity to fill out the survey online. In wave 1, 4,804 immigrants participated. This was a response of 32%, of which the majority filled out the questionnaire by paper and pencil (65%). In the subsequent waves, Statistics Netherlands provided information about movers if they agreed to be re-approached again (97%). Before the second wave in the Spring of 2015, a share of 16% had deregistered from the country's municipality-based registers and were no longer part of the survey population. The panel survey knew a relatively large share of attrition due to the character of the population: recent immigrants form a dynamic population. In particular so the EU populations, who are free to move within the EU. From the approached immigrants in wave 2 (3,847) 59% responded. Similarly, another 11% of the wave 2 population had moved at the start of wave 3, in the Fall of 2016. From the 1,998 approachable immigrants, 68% participated a third time. From the 1,334 respondents who participated in wave 3, another 9% could not be reached in wave 4, because of emigration. A response of 79% led to a final 996 respondents in wave 4. We did not find evidence for selective attrition based on immigrants' self-rated health, which we will describe in the trend of self-rated health. Respondents whose gender or age deviated from wave 1 reports were dropped from the respective wave in which the deviation was found; this was around 5% for each wave.

### Self-Rated Health

In this study we employ the widely used self-rated health (SRH) measurement. Respondents were directly asked to rate their health, with a single question “How would you rate your current health? You can think of both your physical and mental health.” Immigrants could answer “very poor,” “poor,” “average,” “good,” and “very good.” Since this is a general assessment it may encompass many different aspects of health, both physical and psychological. Agyemang et al. (2006) stressed that this single item may be differently interpreted among ethnic or immigrant groups, given the finding that it was associated to chronic illness and health care use conditional on ethnic group. Still, the SRH measurement is widely assessed and seen as a key indicator of immigrant health (Acevedo-Garcia et al., 2010). Immigrants with missing values on self-rated health were not included in the analyses, which equaled to 1.2% in wave 1 and <0.2% in the subsequent waves.

### Economic-Domain Variables

Immigrants were asked about their main activity and whether this concerned being employed or being unemployed. Respondents could also indicate that they were in school as their main activity, pensioned, on care leave, on sick leave, or something else. We coded both “being employed” vs. the rest and the variable “being unemployed” vs. the rest. Another question asked whether immigrants had been unemployed. We will test whether it makes a difference in self-rated health whether immigrants are currently unemployed or whether past unemployment affects self-rated health.

Immigrants' income was assessed by presenting 11 income categories of the net-household income, from which the respondent was asked to pick the one describing the household income best. We disentangled people with a low household income (of below 1500Euro net per month) from the medium and higher income groups. Around 10% of the respondents did not provide an answer; this group was taken as a “missing on income” category.

Among the employed respondents, we use information about their employment relation and employment conditions. We coded whether people with a job had a permanent contract or not. Strained working conditions were assessed by asking about working irregular hours, doing hazardous work or doing physical heavy work. Respondents with a job could indicate whether this applied to them not at all to very often on a 5-point-scale.

### Social-Domain Variables

Contacts in the free time were measured with the questions “How often do you spend time with [country of origin] people in your free time?” and “How often do you spend time with Dutch people in your free time?,” which could be answered with one of the following categories: “every day,” “several times a week,” “a few times a month,” “several times a year,” “less often,” and “never.” We coded the contact variables such, that a high score means more frequent contact. For self-rated health it may be more relevant to know whether people experience loneliness, but no direct questions on loneliness are included in the data. Moreover, immigrants may spend time with people from other origins, which was not assessed either. One of the questions is on the number of people important to the respondent and who the respondent feels close to, living in the Netherlands, not including parents, partners, or children. We coded whether people have someone, or no one, important and close living in the Netherlands, other than parents, partner, or children.

We also assessed whether the respondent had a partner and whether the partner lived in the household or outside the household (if so, most of them abroad). As for children of the respondent, we assessed whether the respondent had a child in the household and whether the respondent had children under 18 in the country of origin.

### Cultural-Domain Variables

Immigrants were asked to what extent they perceive group discrimination, with the question: “Some say that people from [country of origin] are being discriminated against in the Netherlands. How often do you think [country of origin] people are discriminated against in the Netherlands?.” Response categories run from 1 “very often” to 5 “never,” which were recoded so that the highest score refers to strongest perceptions of group discrimination. Perceptions of cultural differences were measured by asking for agreement or disagreement on a five-point scale with the statement that the values of Dutch people and [country of origin people] are irreconcilable. The missing values on the two items together amounted 15% of the immigrants. In analyses including the cultural domain variables, respondents with these missing values were excluded. However, in the models without the cultural domain variables these respondents are included.

### Emotional-Domain Variable: Homesickness

Immigrants were asked directly whether they often feel homesick, to which they could answer with “no, never,” “yes, sometimes,” and “yes, very often.” Missing values on homesickness were deleted throughout all models.

### Control Characteristics

We controlled for gender, age, and the highest level of education obtained in the country of origin. Also, the motives for immigration indicated as “for study” and “for political reasons” and length of stay in months is controlled for. Missing values on the non-nominal control variables were replaced by means.

## Methods

First, we provided the descriptives of the sample and the changes we find in self-rated health. Then we provided evidence to what extent differences between recent immigrants in self-rated health can be attributed to the economic, social, cultural and emotional characteristics. We tested these models within STATA panel modeling, defining the between-effect models (Torres-Reyna, 2007; Statacorp, 2013). The first model included the control characteristics only; the subsequent models included the predictors from the economic domain, social domain, and cultural domain, respectively. The second model including the economic domain indicators was also tested once for the immigrant population who has worked since immigration, in order to account for job-related characteristics. In the fifth models, we also included homesickness (for the model with all immigrants and a model with the immigrants who have worked since immigration). Finally, we employed dynamic models (fixed effect panel models within STATA), testing for differences within immigrants; we tested to what extent changes in the economic, social, cultural, and emotional domain are related to changes in self-rated health over the four waves.

### Analyses

Descriptive statistics among the balanced panel (Table 1) show economic integration in the first years: the share of employed increases from 59% to 73%. Unemployment reflects this trend by decreasing. Also, the share of immigrants with a low income became steadily smaller over time (from 33.3% in wave 1 to 16.7% in wave 4). Among the people who (had) work, an increasing share has a permanent contract, whereas there is mostly stability in job characteristics.

**Table 1:** Descriptive statistics among the balanced panel (*n* = 883).

		**Wave 1**	**Wave 2**	**Wave 3**	**Wave 4**
Good health rating	1–5	4.30	4.18	4.10	3.98
Employed	0/1	59.0	67.4	72.5	73.3
Unemployed at time of interview	0/1	21.5	14.9	11.0	9.0
Been unemployed last year	0/1		34.4	31.8	16.9
Income					
-Medium or high income (ref)	0/1	54.9	49.8	71.3	75.5
-Low income	0/1	33.3	27.2	20.8	16.7
-No information on income	0/1	11.8	10.1	7.8	7.8
Contact with CO people	1–6	4.19	4.23	4.19	4.05
Contact with Dutch	1–6	4.01	3.98	4.10	3.95
Close friend in NL	0/1	91.4	93.0	91.7	94.4
Partner status					
-No partner (ref)		19.5	16.3	15.6	14.4
-Partner in the household		62.2	69.0	71.9	72.8
-Partner outside the household		18.3	14.7	12.5	12.8
Children in the household	0/1	22.4	28.7	33.4	40.4
Children under 18 in CO	0/1	4.5	3.4	3.3	2.8
Homesickness	1–3	2.05	2.06	2.05	2.05
**AMONG RESPONDENTS WITHOUT MISSING VALUES ON CULTURAL VARIABLES (*****N*** **=** **779)**					
Perceived group discrimination	1–5	2.85	2.86	2.89	2.85
Perceived cultural distance	1–5	2.75	2.73	2.69	2.73
**AMONG IMMIGRANTS WHO HAVE (HAD) WORK IN THE NETHERLANDS (*****N*** **=** **640)**					
Permanent contract	0/1	35.6	45.2	57.3	67.8
Working irregular hours	1–5	1.87	1.94	1.96	2.00
Hazardous work	1–5	1.32	1.31	1.36	1.35
Physical heavy work	1–5	1.68	1.68	1.69	1.65

*New Immigrant Survey–the Netherlands−4 waves*.

Contact with country of origin people and Dutch people decreases somewhat in the fourth wave. Perhaps this is due to an increased share of households with a child, which rose from 22% in wave 1 to 40% in wave 4. Also, more respondents live with their partner; an increase from 62% in wave 1 to 73% in wave 4. The average level of perceptions of discrimination and of cultural distance hardly change over time. The level of homesickness was also stable over time (Table 1).

**Figure 1:**
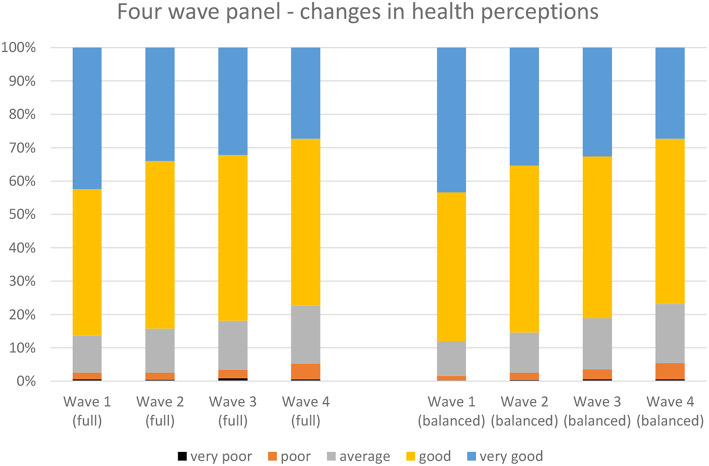
Changes in immigrants' health assessment over the four waves of the panel. **Left:** all immigrants participating in the separate waves. **Right:** balanced panel. New Immigrant Survey–the Netherlands−4 waves.

**Figure 2:**
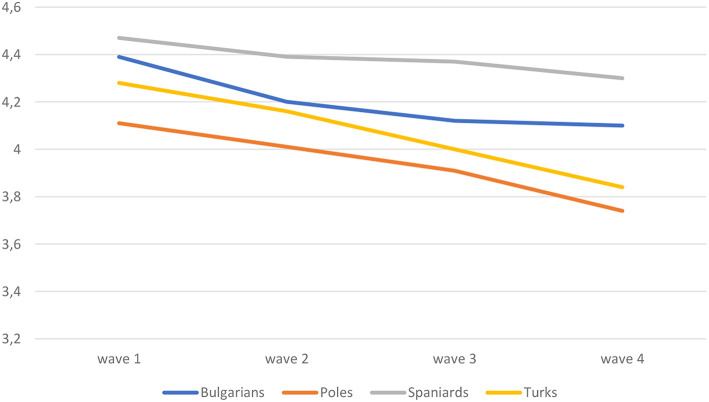
Changes in average self-rated health by immigrant group (balanced panel). New Immigrant Survey–the Netherlands−4 waves.

Figure 1 shows that over the four waves, the self-rated health deteriorates. However, the majority by far rate their health to be good to very good, and this proportion remains on a high level over the four waves of our study. There is, however, a clear tendency that immigrants less often opt for the “very good” health assessment and instead shift to “good health” or “average health.” The share of immigrants rating their health as “poor” is rather small but this increases over time as well. The changes in the left panel of the figure represent the changes among all immigrants who participated in the four waves. Selective return migration and selective panel attrition may, however, have affected this outcome. If the negative health trend is explained by such selection effects, healthier people are particularly likely to return to the country of origin or to drop out, which does not seem very likely. Indeed, when we show the trend only for the immigrants who participated in all four waves (the balanced panel), the pattern is almost identical. We conclude from Figure 1 that immigrants' self-rated health is, in general, positive, however decreasing over time. Figure 2 presents the trend for each of the four immigrant groups. For all of the immigrant groups, the decline in self-rated health is found, although the slope is less steep for the Spanish immigrants. In the remainder of the article we will assess whether differences in self-rated health and the changes therein can be related to and explained by economic, social, cultural, and emotional interpretations of health status. We calculate unstandardized effect sizes, to provide information on what the difference in self-rated health is on the minimum vs. the maximum value of the explanatory variables.

The first model of Table 2 shows that male immigrants rate their health to be better than female immigrants. The older the immigrants are, the lower their health rating. A higher level of education also increases the self-rated health. Immigrants from Spain and Bulgaria rate their health to be better than immigrants from Poland and Turkey; also controlled for educational level and study as motives of migration. Immigrants who came for study rate their health to be better, whereas immigrants who moved for political reasons report worse health. We also find that when immigrants reside longer in the country, their self-rated health decreases, which ties in with the happy immigrant literature: a more positive perspective just after immigration, but a worsening perspective with a longer stay. The effect is not strong though; after a stay of 5 years, the health assessment is estimated to be 0.12 lower (on a five-point scale).

**Table 2:** Recent immigrants' health: between effects, from the economic, social and cultural domain.

	**Model 1**	**Model 2a -all**	**Model 2b -population that has (had) work since immigration**	**Model 3 -all**	**Model 4 -all**
**ECONOMIC**					
Employed		0.09**	0.13**		
*(Unemployed at time of interview)*		*0.03*			
Been unemployed last year		−0.04	−0.04		
Income					
- Medium or high income (ref)					
- Low income		−0.09**	−0.02		
- No information on income		−0.01	0.02		
Permanent contract			0.01		
Irregular work			0.00		
Hazardous work			−0.08**		
Physical heavy work			−0.14***		
**SOCIAL**					
Contact with CO people				0.01	
Contact with Dutch				0.03***	
Relevant and close person in NL				0.13**	
Partner status					
- No partner (ref)					
- Partner in the household				0.03	
- Partner outside the household				−0.03	
Children in the household				−0.05	
Children under 18 in CO				0.08	
**CULTURAL**					
Perceived group discrimination					−0.10***
Perceived cultural distance					−0.04***
**CONTROLS**					
Gender (men)	0.09***	0.06**	0.13***	0.09***	0.09***
Age	−0.01***	−0.01***	−0.01***	−0.01***	−0.01***
Level of education	0.05***	0.04***	0.03***	0.04***	0.04***
Country of Origin					
- Poland (ref)					
- Bulgaria	0.06**	0.09***	0.03	0.05	0.10**
- Turkey	−0.02	0.02	−0.15**	−0.03	−0.04
- Spain	0.15***	0.14***	0.03	0.13***	0.08*
Reason of immigration: Study	0.09**	0.12***	−0.02	0.07*	0.05
Reasons of immigration: Political	−0.20***	−0.20***	−0.14**	−0.20***	−0.18***
Months since immigration	−0.002***	−0.002***	−0.002**	−0.002***	−0.001***
Number of respondents	4, 734	4, 734	3, 519	4, 734	4, 169
Number of observations	8, 987	8, 987	6, 211	8, 987	7, 589

*** *p < 0.001*,

** *p < 0.01*,

* *p < 0.05*.

*New Immigrant Survey–the Netherlands−4 waves*.

In the economic domain, being employed is related to better self-rated health, but its effect is limited; not larger than 0.09 among all immigrants, and 0.13 when the sample is restricted to immigrants who did work since immigration (Table 2, models 2a and 2b). Surprisingly, unemployment does not reduce the self-rated health significantly (neither current unemployment nor having been unemployed in the last year). Immigrants with a low income rate their health to be poorer as compared to immigrants with a medium to high level income; however, here the effect is limited in size. On the five-point-scale, the lower income group rates the health 0.09 lower.

Among the immigrants who (had) work, a permanent job position is not associated to self-rated health. More hazardous work reduces good health rating (*b* = −0.08) and this holds even stronger for doing physically heavy work (*b* = −0.14). Immigrants performing physically heavy work rate their health 0.56 lower than immigrants not doing so. The effect of income is interpreted by the job-characteristics; it is thus not so much the lower income that reduces self-rated health, but the more hazardous work and physically heavy work that characterizes low income jobs.

In the social domain, model 3 in Table 2 shows that immigrants having more contact with Dutch people report better health, and so do immigrants reporting to have at least one relevant and close person to them living in the Netherlands. The frequency of contact with country of origin people, or having a partner or children in the household or living in the country of origin itself are not associated to self-rated health.

Model 4 in Table 2 provides evidence that recent immigrants who perceive that their immigrant group is more often discriminated against in the Netherlands rate themselves to be less healthy; immigrants who perceive discrimination very often score 0.40 lower than immigrants perceiving no discrimination at all. This lower health rating also holds for immigrants who perceive a stronger incompatibility between the Dutch culture and the country of origin culture; but the effect is smaller (b=-0.04).

In models 5a and 5b, presented in Table 3, we show evidence for the role of homesickness. The effect of homesickness turns out to be relevant in understanding recent immigrants' self-rated health. The more often immigrants report homesickness, the lower their self-rated health. The difference between immigrants never experiencing homesickness and those experiencing it often is 0.24. In this last model, the effect of perception of cultural distance is no longer significant, implying that the relation between cultural distance and self-rated health can be interpreted by homesickness. This partly holds for perceptions of discrimination as well, although the effect of perceived discrimination remains significant once homesickness is included. The other effects in the model are hardly affected by the inclusion of homesickness.

**Table 3:** Recent immigrants' health: between effects, including homesickness.

	**Model 5a -all**	**Model 5b -population that has (had) work since immigration**
**ECONOMIC**		
Employed	0.10**	0.09*
Been unemployed last year	−0.02	−0.04
Income		
- Medium or high income (ref)		
- Low income	−0.05	−0.01
- No information on income	0.02	0.01
Permanent contract		−0.01
Irregular work		0.00
Hazardous work		−0.07**
Physical heavy work		−0.15***
**SOCIAL**		
Contact with CO people	0.02*	0.02*
Contact with Dutch	0.02**	0.03**
Relevant and close person in NL	0.12**	0.11*
Partner status		
- No partner (ref)		
- Partner in the household	0.05	0.05
- Partner outside the household	−0.01	0.02
Children in the household	−0.08**	−0.06
Children under 18 in CO	0.07	0.02
**CULTURAL**		
Perceived group discrimination	−0.09***	−0.08***
Perceived cultural distance	−0.03**	−0.03*
**EMOTIONAL**		
Homesickness	−0.12***	−0.09***
**CONTROLS**		
Gender (men)	0.05*	0.12***
Age	−0.01***	−0.01***
Level of education	0.03***	0.02**
Country of Origin		
- Poland (ref)		
- Bulgaria	0.12***	0.07
- Turkey	−0.01	−0.12**
- Spain	0.08*	−0.02
Reason of immigration: Study	0.06	−0.03
Reasons of immigration: Political	−0.19***	−0.15**
Months since immigration	−0.002***	−0.001*
Number of respondents	4, 169	3, 143
Number of observations	7, 589	5, 357

*** *p < 0.001*,

** *p < 0.01*,

* *p < 0.05*.

*New Immigrant Survey–the Netherlands−4 waves*.

Model 5b from Table 3 has also been tested for each of the four immigrant groups separately. Appendix 1 presents the findings. For each of the immigrant groups, physical heavy work is associated with lower health. Social contact with Dutch is positive among all groups but reaches significance among these four smaller samples only among the Turkish immigrants. Discrimination perceptions are consistently related to lower health ratings across the immigrant groups. Homesickness only reaches significance among Polish and Spanish immigrant groups.

#### Dynamic Models

Table 4 presents the findings from the dynamic fixed-effect models. It shows to what extent changes within recent immigrants come together with changes in self-rated health in model 1a, the model for all immigrants, and in model 1b, the findings for the immigrants who have worked since immigration. Findings are, overall, similar in the two models. In a model without predictors, Rho (intraclass correlation coefficient) equals 0.62, which is the proportion of variance in rated health due to differences between immigrants; the within person variance is 0.38.

**Table 4:** Recent immigrants' health: fixed effects (within individuals).

	**Model 1a -all**	**Model 1b -population that has (had) work since immigration**
**ECONOMIC**		
Employed	−0.01	−0.03
Been unemployed last year	−0.02	−0.04
INCOME		
- Medium or high income (ref)		
- Low income	0.06	0.05
- No information on income	0.06	−0.04
Permanent contract		−0.04
Working irregular hours		0.02
Hazardous work		−0.06*
Physical heavy work		−0.05*
**SOCIAL**		
Contact with CO people	0.00	−0.01
Contact with Dutch	0.02**	0.03*
Relevant and close person in NL	−0.02	0.06
Partner status		
- No partner (ref)		
- Partner in the household	−0.03	−0.05
- Partner outside the household	−0.06	−0.07
Children in the household	−0.11**	−0.07
Children under 18 in CO	0.06	0.05
**CULTURAL**		
Perceived discrimination	−0.04**	−0.08***
Perceived cultural distance	−0.04**	−0.02
**EMOTIONAL**		
Homesickness	−0.03	−0.05
Number of respondents	4, 169	3, 143
Number of observations	7, 589	5, 357

*** *p < 0.001*,

** *p < 0.01*,

* *p < 0.05*.

*New Immigrant Survey–the Netherlands-4 waves*.

Strikingly, neither a change in paid work nor in income affects changes in self-rated health. From the economic domain, we do find small effects of changes in hazardous work and physical heavy work. Immigrants with a job who report over time that their job has become more hazardous or more physically heavy decrease in their self-rated health.

An increase in contacts with Dutch residents is associated with an increase in self-rated health. The birth of a child in the household slightly decreases self-rated health (*b* = −0.11), but not significantly so within the restricted sample of the working population (Table 4, model 1b). Finally, we find that immigrants who perceive an increase in group discrimination and an increase in perceived cultural distance during these first years after immigration rate their health poorer over time. Among the population that had paid work since immigration, it is mainly the change in perceived discrimination that contributes to a lower self-rated health, with a maximum decrease of 0.32 when immigrants change from the perception that there is no discrimination at all, to the perception that their country of origin group is discriminated against often. Whereas homesickness explained why there are differences in self-rated health between immigrants, *changing* homesickness is not associated with changes in self-rated health.

Appendix 2 presents the fixed-effect models for each of the immigrant groups. Samples for the immigrant groups are relatively small (in particular so for the Bulgarian and Turkish immigrants), implying that effects reach significance less easily. Hazardous work and physically heavy work affect self-rated health only among Spaniards and Turks, respectively. Changes in contacts with Dutch are positive among all groups, but significant only among Turkish immigrants. Increasing perception of discrimination is significantly related to lower rating of health for all groups but the Bulgarians. Among this latter group an increase in perceived cultural distance as well as homesickness is related to a decrease in health rating.

## Conclusions And Discussion

Immigrants deliberately moving immigrate in search for a better income, to join a partner, to start a study, or just opt for a better life in another country, and mostly perceive their health as good, or very good. A small 2% of the recent immigrants assessed their health status as (very) poor. In contrast, 43% of the respondents of our panel stated their health to be very good. Also, after the 5 years in which the immigrants participated in the survey for the first time, the self-rated health among the vast majority is good. The proportion of immigrants assessing their health as (very) poor increases, but still, only 5% do so. The major change is shown by the decrease in the share of immigrants reporting “very good” health. It steadily declined from 43% in the first approach to 27% in the fourth wave.

What is associated with a lower health reporting? We have shown that immigrants in hazardous and physically heavy work conditions rate their health to be lower, explaining why immigrants with lower income have a lower self-rated health. Immigrants being in a paid job show a very limitedly better self-rated health, whereas unemployment does not decrease health assessment. Also, a more secure labor market position, expressed by a permanent contract, does not make a difference in self-rated health. In the social domain, immigrants with more contacts show better health. Most convincing, though, are cultural and emotional explanations: those who perceive discrimination, cultural distance, and home-sickness report lower health.

Now, which changes were associated with lower health assessment during the four waves? Strikingly, changes in the objective economic conditions did not change immigrant's self-rated health. Immigrants who obtained a job, and changed status from unemployed to employed, did not report a change in their health. Also, a change to a higher income or obtaining a permanent job was not associated with lower self-rated health. We do find evidence that evaluation of the work becoming more hazardous or more physically heavy is related to lower self-rated health.

In the social domain, an increase in contacts with the Dutch is associated with better health. Over time, the frequency of contacts did not increase on average and, hence, cannot explain the downward trend in health rating. We found that a first-born in the household is associated with a decrease in self-rated health. Since this is one of the major changes in the period in which the immigrant population is studied here, it may account for the less positive health rating over the years we followed the recent immigrants. Recent immigrants with first-borns can rely less on family for informal support and care-arrangements, which may make it particularly hard for immigrants to combine family life and work. Immigrants in the Netherlands have been found to be rather critical of the (costly) child-care (Lubbers et al., 2018a); once immigrants have children they may experience the high costs of child care as an additional burden on their household, possibly increasing tensions.

Homesickness hardly changed over the course in which immigrants were studied and we did not find evidence that increased homesickness within respondents was associated with lower reported health. We do find that an increase in perceptions of cultural distance, between the Dutch culture and country of origin culture, is associated with lower health, but in particular that an increase in perceived group discrimination is associated with lower health. Still, over the four waves, there are no marked increases in both perceptions of cultural distance or perceived discrimination that can explain the less positive health assessment over time.

McDonald and Kennedy (2004) rightly claimed that more research is needed into the interpretation of good and bad health or, in this case, perhaps more relevant research of good and very good health. Immigrants may have perceived themselves to be in very good health on arrival but may consider themselves, as compared to the lifestyles of the healthier part of the receiving population, not as healthy as initially thought. It is an interesting question to whom immigrants compare themselves, when evaluating their health. Another way to increase insight in declining health assessment would be to study the combination of changes in the economic, social, and cultural domains with the adoption (or acculturation) of more unhealthy lifestyles (Antecol and Bedard, 2006); i.e., more stress, larger intake of calories, higher alcohol consumption and, perhaps relevant in the Dutch context, other drug usage. Although there is much attention in the international literature on self-rated health, which has shown that self-rated health is a relevant predictor for health outcomes, it is unfortunate that we were not able to test whether the explanatory model we tested here also holds true for other health outcomes.

Another promising direction is to focus on immigrants' unmet expectations, such as has been found to contribute to frustrations in health care among Somali in the US (Pavlish et al., 2010) and Sudanese in Canada (Simich et al., 2006). We found that homesickness did not increase over time. Instead, a more specific assessment of decreasing satisfaction with life in the host country could be the key to understanding decreasing health rating among immigrants in the first years after immigration. What stands out though, is that subjective evaluations of immigrants' situations are key to understanding how they rate their health. The strongest role we found is that for perceived discrimination. Immigrants becoming aware of ethnic group discrimination decline in self-rated health.

## Ethics Statement

The data collection for this project started in 2013. An ethics approval was not required as per applicable institutional and national guidelines and regulations at the time. The design of the data collection was approved by the Dutch Science Foundation. Sampling of respondents is conducted by Statistics Netherlands. Statistics Netherlands also approved the letters sent to respondents to request their participation.

## Author Contributions

All authors listed have made a substantial, direct and intellectual contribution to the work, and approved it for publication.

### Conflict of Interest Statement

The authors declare that the research was conducted in the absence of any commercial or financial relationships that could be construed as a potential conflict of interest. The reviewer ES declared a past co-authorship with one of the authors ML to the handling editor.
