# Supplementary material for: Changes in Self-Rated Health Right After Immigration: A Panel Study of Economic, Social, Cultural, and Emotional Explanations of Self-Rated Health Among Immigrants in the Netherlands
Source: Front Sociol. 2019 Jun 6;4:45. doi: 10.3389/fsoc.2019.00045 (PMC8022797; doi:10.3389/fsoc.2019.00045)
Supplement: Supplementary file 1 [file Data_Sheet_1.pdf]

Appendix 1. Recent immigrants' health: between effects, by immigrant group

	Poles	Bulgarian	Spaniards	Turks
**Economic**				
Employed	.09	.01	.14*	.06
Been unemployed last year	-.02	-.14	.09	.00
Income				
- Medium or high income				
- Low income	-.04	.07	.04	-.13
- No information on income	-.13	.12	.10	.01
Fixed contract	-.03	-.06	-.01	.14
Irregular work	-.01	.07	-.03	-.02
Hazardous work	-.11**	-.06	.02	.03
Physical heavy work	-.17***	-.20**	-.08*	-.29***
**Social**				
Contact with co people	.03*	.06*	.02	-.01
Contact with Dutch	.02	.03	.02	.06*
Relevant and close person in NL	.14*	-.12	.04	.28
Partner status				
- No Partner (ref)				
- Partner in the household	-.01	.09	.13*	.09
- Partner outside the household	.01	.05	-.01	.00
Children in the household	-.06	-.05	-.13~	.06
Children under 18 in CO	-.01	.04	-.10	.45~
**Cultural**				
Perceived group discrimination	-.05*	-.07~	-.07**	-.19***
Perceived cultural distance	-.01	-.07~	-.04~	-.04
**Emotional**				
Homesickness	-.11***	-.09	-.11**	.02
Controls				
Gender (men)	.18***	.17*	.01	.16
Age	-.01***	-.02***	-.01	-.03***
Level of education	.03*	.05*	.03*	-.00
Reason of immigration: Study	.05	-.19~	-.04	-.03
Reasons of immigration: Political	-.24**	.19	-.09	-.50*
Months since immigration	-.000	.002	-.001	-.000
Number of respondents	1427	392	948	376
Number of observations	2340	617	1747	653

**** p < .001; ** p < .01; * p < .05*

Source: New Immigrant Survey – the Netherlands – 4 waves

Appendix 2. Recent immigrants' health: fixed effects, by immigrant group

	Poles	Bulgarian	Spaniards	Turks
**Economic**				
Employed	-.01	-.24~	.03	.11
Been unemployed last year	-.07	.08	.03	.04
Income				
- Medium or high income				
- Low income	.07	-.15~	.12	-.01
- No information on income	-.07	-.10	-.03	-.05
Fixed contract	-.05	-.01	-.08	.00
Irregular work	-.01	.06	.06~	.02
Hazardous work	-.03	.06	-.12*	-.09
Physical heavy work	-.04	.00	.01	-.24**
**Social**				
Contact with co people	-.02	.01	-.01	.02
Contact with Dutch	.02	.08**	.01	.03
Relevant and close person in NL	-.11	.17	.06	-.25
Partner status				
- No Partner (ref)				
- Partner in the household	-.05	-.14	-.07	.08
- Partner outside the household	-.04	-.21	-.09	-.10
Children in the household	-.13~	.05	-.02	-.06
Children under 18 in CO	-.04	.13	-.00	.68~
**Cultural**				
Perceived group discrimination	-.10**	.05	-.07*	-.12*
Perceived cultural distance	-.00	-.09*	-.03	.00
**Emotional**				
Homesickness	-.06	-.17*	-.05	.03
Number of respondents	1427	392	948	376
Number of observations	2340	617	1747	653

**** p < .001; ** p < .01; * p < .05*

Source: New Immigrant Survey – the Netherlands – 4 waves
